# Assessing fidelity of delivery of smoking cessation behavioural support in practice

**DOI:** 10.1186/1748-5908-8-40

**Published:** 2013-04-04

**Authors:** Fabiana Lorencatto, Robert West, Charlotte Christopherson, Susan Michie

**Affiliations:** 1 NHS Centre for Smoking Cessation and Training, Dept. Clinical, Educational & Health Psychology, University College London, London, WC1E 7HB, UK; 2 Centre for Outcomes Research and Effectiveness, Dept. Clinical, Educational & Health Psychology, University College London, London, WC1E 7HB, UK; 3 CRUK Health Behaviour Research Centre, Dept. Epidemiology & Public Health, University College London, London, WC1E 7HB, UK; 4 Department of Psychology and Language Sciences, University College London, London, WC1E 7HB, UK

**Keywords:** Behaviour change interventions, Smoking cessation, Delivery, Fidelity, Implementation

## Abstract

**Background:**

Effectiveness of evidence-based behaviour change interventions is likely to be undermined by failure to deliver interventions as planned. Behavioural support for smoking cessation can be a highly cost-effective, life-saving intervention. However, in practice, outcomes are highly variable. Part of this may be due to variability in fidelity of intervention implementation. To date, there have been no published studies on this. The present study aimed to: evaluate a method for assessing fidelity of behavioural support; assess fidelity of delivery in two English Stop-Smoking Services; and compare the extent of fidelity according to session types, duration, individual practitioners, and component behaviour change techniques (BCTs).

**Methods:**

Treatment manuals and transcripts of 34 audio-recorded behavioural support sessions were obtained from two Stop-Smoking Services and coded into component BCTs using a taxonomy of 43 BCTs. Inter-rater reliability was assessed using percentage agreement. Fidelity was assessed by examining the proportion of BCTs specified in the manuals that were delivered in individual sessions. This was assessed by session type (*i.e*., pre-quit, quit, post-quit), duration, individual practitioner, and BCT.

**Results:**

Inter-coder reliability was high (87.1%). On average, 66% of manual-specified BCTs were delivered per session (SD 15.3, range: 35% to 90%). In Service 1, average fidelity was highest for post-quit sessions (69%) and lowest for pre-quit (58%). In Service 2, fidelity was highest for quit-day (81%) and lowest for post-quit sessions (56%). Session duration was not significantly correlated with fidelity. Individual practitioner fidelity ranged from 55% to 78%. Individual manual-specified BCTs were delivered on average 63% of the time (SD 28.5, range: 0 to 100%).

**Conclusions:**

The extent to which smoking cessation behavioural support is delivered as specified in treatment manuals can be reliably assessed using transcripts of audiotaped sessions. This allows the investigation of the implementation of evidence-based practice in relation to smoking cessation, a first step in designing interventions to improve it. There are grounds for believing that fidelity in the English Stop-Smoking Services may be low and that routine monitoring is warranted.

## Introduction

Behavioural support for smoking cessation can be a highly cost-effective, life-preserving intervention [1-3]. It consists of advice, discussion, and targeted activities designed to minimize smokers’ motivation to smoke, maximize resolve not to smoke, help with strategies to minimize exposure to smoking cues, cope with urges when they occur, and make best use of adjunctive activities, such as smoking cessation medications [4-6]. With the growing emphasis on promoting evidence-based practice, behavioural support interventions shown to be effective in research trials have been increasingly implemented as part of routine healthcare practice in numerous high and middle income countries [7]. For instance, in the UK, implementation is via a network of locally organized Stop-Smoking Services, which offer smokers who are trying to quit medication and, typically, four free, weekly behavioural support sessions. Smokers engaging with these services are on average four times more likely to quit [8].

The translation of clinical research findings into practice is not straightforward, and is often slow and unpredictable [9]. Methods are needed to promote the consistent, systematic uptake of research findings concerning the evidence-base of behaviour change interventions into routine practice [9]. Treatment manuals represent one potential vehicle by which the content of interventions with demonstrated effectiveness may be translated into the content of clinical practice. The term ‘treatment manual’ typically refers to structured, procedural books outlining the rationale and goals of an intervention, as well as the recommended content (*i.e*., behaviour change techniques) to be delivered when administering an intervention [10]. Use of manuals offer numerous advantages for clinical practice; they said the dissemination and replication of interventions, make the content of time-limited interventions more structured and focused than they might be otherwise, and facilitate training and supervision of intervention providers [10,11]. The recent increase in the pressure to employ treatment manuals has extended beyond controlled research trials into practice, and evidence is emerging, supporting the use of manuals in clinical practice [11,12].

Treatment manuals are widely used in the delivery of smoking cessation behavioural support interventions. In the UK, national guidelines outlining the recommended content and format of smoking cessation behavioural support sessions have been published [13]. These recommend that evidence-based guidelines [14] should inform how behavioural support is delivered by the English Stop-Smoking Services. Most of these services have a treatment manual providing standardized guidance for practitioners regarding the specific content to be delivered in different types of behavioural support sessions (*i.e*., pre-quit, quit-day and post-quit). However, there is evidence that different stop-smoking practitioners providing support in English Stop-Smoking Services and operating to the same treatment manual can have widely differing success rates [15]. This raises an important question as to how far behavioural support is delivered according to specification in treatment manuals, and whether practitioners are adhering to, or deviating from, manual-based treatment specifications. This paper reports an evaluation of a method for assessing this and preliminary results of its application in routine clinical practice.

Fidelity of intervention delivery refers to the extent to which interventions are delivered as intended, with adherence to specifications in intervention manuals [16,17]. It specifically concerns whether core, prescribed intervention components are delivered, rather than the separate but associated question of how components are delivered, for example, in terms of quality or tailoring of delivery. Assessing fidelity of delivery is part of the continuous assessment, monitoring and improvement of the reliability and internal validity of an intervention [16]. Verifying the extent to which intervention content is delivered according to manual specification is critical for the accurate interpretation of intervention outcomes [16,18]. Assessing fidelity can also highlight both provider training needs and aspects of intervention delivery that require improvement. The need to examine fidelity has been underlined in the CONSORT statement for reporting complex, non-pharmacological interventions [19].

Although the importance of examining fidelity of delivery is widely recognised, reviews to date suggest that it is not frequently assessed, reported, or accounted for in analyses [16,18,20,21]. To date, research efforts have primarily focused on the development and evaluation of new interventions rather than monitoring and improving the fidelity with which interventions are delivered when subsequently implemented in practice [9]. Recommendations of methods for assessing fidelity are widely available [16-18], but these are rarely applied. Recently developed methods for assessing the fidelity of delivery of behaviour change interventions for physical activity [22] and excessive alcohol use [23] use the recommended ‘gold standard’ strategy of objectively verifying delivery by comparing the content of recorded intervention sessions to pre-specified criteria, such as an intervention manual [16]. Where fidelity of delivery has been assessed, it is often found to be poor (<55%) and rarely uniform [18,20-23]. There is currently no standard method for assessing fidelity of delivery of smoking cessation behaviour change interventions.

The recent development of a theory-linked taxonomy of 43 smoking cessation BCTs has provided a reliable method for specifying the content of behavioural support interventions in terms of their component BCTs [24]. Each BCT has specified criteria for its operationalization, is defined using consistent terminology, and has a clear label that can be used to categorize and consistently report intervention components. A total of 14 BCTs from the taxonomy have been supported by RCT evidence, and 16 have been shown to be significantly associated with improved four-week CO-validated quit outcomes [25,26]. This taxonomy has been reliably applied in a previous study as a coding framework for identifying and categorizing component BCTs present in English Stop-Smoking Service treatment manuals [4,6,25] and transcripts of audio-recorded behavioural support sessions delivered by these services [27]. However, the taxonomy has not yet been used to compare the content of treatment manuals with the transcripts of corresponding behavioural support sessions to assess fidelity.

This study aimed to evaluate the taxonomy as a method for investigating variations in the fidelity of delivery of smoking cessation behavioural support delivered in two English Stop-Smoking Services. In addition to examining the extent to which manual-specified content is delivered, this study was designed to investigate delivery of BCTs not specified in manuals. Examining additional content is important, as such content introduces further variability in practice and outcomes. Additional content may augment or detract from manual-specified content.

The specific objectives of this study were:

1. To evaluate a method of assessing fidelity of behavioural support for smoking cessation using a taxonomy of behaviour change techniques;

2. To assess using this method the fidelity of delivery of behavioural support in two English Stop-Smoking Services;

3. To examine variation in fidelity according to: session type (*i.e*., pre-quit, quit-day, post-quit); session duration; stop smoking practitioner; and the specific BCT;

4. To assess the extent of use of BCTs not included in the particular treatment manual in operation.

## Methods

### Ethical approval

This study received ethical approval by the Clinical, Educational, and Health Psychology Research Department Ethics Committee (UCL) [Reference: CEHP/2011/038].

### Design

This observational study assessed fidelity of delivery by comparing the content, in terms of component BCTs, of treatment manuals with the corresponding transcripts of audio-recorded behavioural support sessions.

### Study sample and materials

Data were obtained from two English Stop-Smoking services, which typically offer medication and four weekly behavioural support sessions. Behavioural support is typically provided by trained, specialist advisors, often of multidisciplinary backgrounds (*i.e*., nurses, midwives, GPs, pharmacists). The first session is typically a ‘pre-quit session,’ which aims to enhance a smoker’s motivation and self-confidence to quit, set clear goals, discuss medication options, and address general preparations for quitting. The second session is the ‘quit-day’ session, which focuses on general strategies for avoiding smoking cues and overcoming barriers to cessation, as well as maintaining motivation and self-efficacy. The final two sessions are post-quit sessions, which concentrate on equipping the client with strategies for avoiding smoking in the long term by facilitating relapse prevention and coping, alongside promoting an ex-smoker identity. Service 1 is based in the north of England and has the highest CO-validated four-week quit rate of 59% (April to December 2011). Service 2 is based in North East London, UK, and has an average CO-validated four-week quit rate of 38% (April to December 2011). The average CO-validated quit rate in the Stop-Smoking Services in April to December 2011 was 35%, range 5% to 59%) [28].

The treatment manual was obtained from each service. A treatment manual was defined as any guidance document providing a ‘formal, written plan specifying procedures to be followed in providing a specific treatment or support for smoking cessation to smokers’ [6]. Manuals are usually written in-house by each service and typically outline the specific content to be delivered by practitioners in either a pre-quit, quit-day or post-quit behavioural support session. Manuals therefore represent ‘recommended’ practice, and in theory incorporate national guidance and training standards [13,29].

Audio recordings of consecutive behavioural support sessions delivered to consenting clients as part of routine clinical practice were obtained during a two-month data collection period. This minimized the opportunity for practitioners to select which clients to record. The resulting sample comprised 30 recordings from Service 1, and 13 recordings from Service 2. Nine audio recordings from Service 1 were excluded from analysis as they were incomplete. A mixture of session types (pre-quit, quit day, and post-quit) were audio recorded by the practitioner using a discrete recording device. Of the 21 usable recordings from Service 1*,* 4 were of pre-quit sessions, 2 quit-day, and 15 post-quit. For Service 2, 4 recordings were of pre-quit sessions, 2 quit-day, and 7 post-quit. All audio recordings were anonymized and transcribed verbatim.

### Procedure

Informed consent to audio recorded sessions and having session content examined by research psychologists was obtained from the practitioner and client. Coding was conducted by two research psychologists (researcher initials: FL, CC) with previous training and experience in coding using the taxonomy. Both researchers independently coded all study materials (*i.e*., 2 manuals, 34 transcripts). The treatment manuals were coded into component BCTs using an established taxonomy of 43 smoking cessation BCTs with demonstrated reliability for coding service treatment manuals [4,6,24,25]. Content of treatment manuals was coded according to content pertaining to either pre-quit, quit-day, or post-quit support. Transcripts of audio-recorded behavioural support sessions were coded into component BCTs using a recently adapted taxonomy of 44 smoking cessation BCTs with demonstrated reliability for coding transcripts of audio-recorded behavioural support sessions delivered by Stop-Smoking Services [27]. This adapted taxonomy is an updated version of the original taxonomy of 43 BCTs. Adaptations included merging typically co-occurring BCTs and refining existing BCT labels and definitions [27]. The resulting content of the taxonomies is therefore largely comparable and comprises the same BCTs.

If coders identified the same BCT within a section of text, agreement was registered. Where one coder identified a BCT and the other did not, or a different BCT was identified, disagreement was registered. If an intervention component could not be coded by a BCT label from the taxonomy, this was identified as a potential new BCT. Discrepancies were resolved through discussion or consultation with a behaviour change expert (SM).

### Analyses

Inter-rater coding reliability was assessed by examining the proportion of all BCTs identified within a transcript that were identified by both coders (*i.e*., % positive agreement). Percentage agreement was used rather than Cohen’s Kappa for numerous reasons. First, the items being coded (*i.e*., sentences within transcripts) were not mutually exclusive, as multiple BCTs may be present within a single sentence. Secondly, BCTs may occur multiple times within a single transcript, with coders potentially agreeing in one instance within the transcript that the BCT is present, but not in another. This does not allow a global present/absent rating for the entire transcript for each BCT. Furthermore, given the high number of 43 BCTs, the probability of selecting a particular code by chance is low. Since Kappa corrects for chance agreement amongst multiple coders, use of Kappa is likely to underestimate reliability [30].

The proportion of BCTs specified in service treatment manuals that were delivered in practice was examined according to session type rather than overall, as both services’ treatment manuals had individual sections pertaining to either pre-quit, quit-day or post-quit support, and BCTs did not feature uniformly across all three sections of each manual. Fidelity of delivery for pre-quit sessions was assessed by examining the proportion of BCTs specified in the pre-quit section of the manual that were delivered in pre-quit behavioural support sessions. This was repeated for quit-day and post-quit sessions, and levels of fidelity compared across session types. These analyses were done both separately and combined across services.

The association between session duration and the proportion of manual-specified BCTs delivered with fidelity was examined by means of Pearson correlations. This analysis was done separately and combined across services.

The mean proportion of manual-specified BCTs delivered by individual practitioners across sessions was calculated for each practitioner and compared across practitioners within each service.

For each manual-specified BCT, fidelity of delivery was assessed by establishing the proportion of sessions each BCT was delivered in according to manual-specification. This was first done according to session type then combined across session types and services, as not all BCTs featured consistently across all three sections of the manual.

The proportion of all BCTs delivered within each session that were not specified by the manual was also calculated.

## Results

### 1. Reliability of fidelity assessment method

Mean inter-rater reliability for coding was 87.1% agreement across transcripts from both services, which is high (*i.e*., > 75%). Mean agreement for Service 1 was 80.9% (range 70.9% to 93.7%), and for Service 2, 93.4% (78.4% to 95.6%).

### 2. Overall fidelity of delivery in two NHS stop-smoking services

In Service 1, across all transcripts, the mean proportion of manual-specified BCTs delivered was 66.4% (SD 16.0; range: 38% to 90%). The average for Service 2 was 65.5% (SD 14.5; range: 35% to 85%) (Additional file 1).

### 3. Variation in fidelity of delivery

#### (i) According to session type

The number of BCTs identified in the pre-quit, quit day and post-quit sections of each service’s treatment manual is provided in Table 1. A full list of BCTs identified within each section of the manual is available in Additional file 2. The mean number (%) of manual-specified BCTs delivered in each session (*i.e*., % fidelity) is presented according to session type, by service, in Table 1. This, alongside general session characteristics, is available for each of the 34 individual transcripts in Additional file 1.

**Table 1:** Summary of mean session characteristics and the proportion of BCTs specified in the treatment manuals delivered individual behavioural support sessions; presented by Stop-Smoking Service and according to session type

**Service**	**Session type (No. of Transcripts)**	**Mean session duration (Min.Sec) (SD)**	**Number of BCTs in manual (according to session type)**	**Mean number of manual specified BCTs delivered (%) (Range)**	**Mean total number of BCTs delivered (SD)**	**Mean number of non-manual specified BCTs delivered (% of total) (Range)**
Service 1	Pre-Quit (4)	28.59 (SD 5.95)	13 -	7.5 (58%) (R: 38% to 69%)	22 (SD 3.94)	14.5 (66%) (R: 47% to 75%)
Service 1	Quit-day (2)	26.41 (SD 2.72)	8 -	5 (63%) (R: 50% to 75%)	23 (SD 3.94)	18 (78%) (R: 78% to 79%)
Service 1	Post-Quit (15)	11.73 (SD 2.72)	10 -	7 (69%) (R: 40% to 90%)	19 (SD 3.94)	12 (63%) (R: 34% to 82%)
Service 2	Pre-Quit (4)	12.62 (SD 5.26)	12 -	9 (75%) (R: 67% to 83%)	23 (SD. 3.55)	14 (61%) (R: 44% to 69%)
Service 2	Quit-day (2)	16.66 (SD 4.96)	21 -	17 (81%) (R: 76% to 85%)	29 (SD 2.82)	12 (41%) (R: 41% to 42%)
Service 2	Post-Quit (7)	11.04 (SD 4.33)	17 -	9.6 (56%) (R: 35% to 64%)	20 (SD 3.8)	10.4 (52%) (R: 45% to 69%)

Across both sets of transcripts, the mean proportion of manual-specified BCTs delivered per session was 66% (SD 14; range: 38% to 83%) for pre-quit sessions, 72% (SD 15.01; range: 50% to 85%) for quit-day sessions, and 62% (SD 16.4, range: 35% to 90%) for post-quit sessions (Table 1; Additional file 1).

In Service 1, fidelity was on average highest for post-quit sessions, with a mean of 69% of manual-specified BCTs delivered per post-quit session, and lowest for pre-quit sessions (mean 58%) (Table 1). In Service 2, fidelity was on average highest in quit-day sessions (mean 81%) and lowest in post-quit sessions (56%) (Table 1).

#### ii) As a function of session duration

Sessions lasted a mean of 15.58 minutes (SD 8.4; range: 5.01 to 36.36) and 12.39 minutes (SD 4.7; range: 5.17 to 20.17) for Service 1 and Service 2, respectively (Table 1; Additional file 1: Table S1). There was no significant correlation between session duration and the proportion of manual-specified BCTs delivered with fidelity in Service 1 (r = 0.122, p = 0.599), Service 2 (r = 0.443, p = 0.129), or across both services (r = 0.17, p = 0.923).

#### iii) According to stop-smoking practitioner

Behavioural support sessions in Service 1 were delivered by five practitioners, each delivering on a mean of 4.2 sessions (range: 3 to 6). The mean proportion of manual-specified BCTs delivered by each practitioner was 67% (SD 9.3) across session types, ranging from 55% to 78%. (Additional file 1). Behavioural support sessions in Service 2 were delivered by four practitioners, each delivering a mean of 3.25 sessions (range: 2 to 4). On average, each practitioner delivered 67.4% (6.5) of manual-specified BCTs across session types, ranging from 58% to 74% (Additional file 1).

#### iv) By specific BCT

Across both services, each manual-specified BCT was delivered according to manual specification in 63% of sessions (SD: 28.5, range 0% to 100%). BCTs for which fidelity of delivery was 100% included: ‘boost motivation and self-efficacy,’ ‘strengthen ex-smoker identity,’ ‘advise on avoidance of cues for smoking,’ and ‘information gathering and assessment.’ Fidelity was lowest for BCTs: ‘set graded tasks’ (0%), ‘prompt commitment from the client there and then’ (15%), ‘advise on/facilitate use of social support’ (15%), and ‘offer/direct towards appropriate written materials’ (28%) (Table 2). The proportion of sessions in which individual manual-specified BCTs were delivered with fidelity according to session type across both services is available in Additional file 3.

**Table 2:** Number of behavioural support sessions in which each BCT was delivered according to manual specification across both services

**BCT label**	**Number of sessions BCT delivered in according to manual (max 34)**
1. Provide information on the consequences of smoking and smoking cessation	4/7 (57%)
2. Boost motivation and self-efficacy	2/2 (100%)
3. Provide rewards contingent on successfully stopping smoking	13/22 (59%)
4. Provide rewards contingent on effort or progress	18/22 (82%)
5. Prompt commitment from the client there and then	2/13 (15%)
6. Strengthen ex-smoker identity	2/2 (100%)
7. Identify reasons for wanting and not wanting to stop smoking	9/13 (69%)
8. Measure carbon monoxide (CO) and explain the purpose of CO monitoring	30/34 (88%)
9. Distract from motivation to engage in behaviour	1/2 (50%)
10. Facilitate barrier identification and problem solving	6/9 (67%)
11. Facilitate relapse prevention and coping	7/13 (54%)
12. Facilitate action planning/ develop treatment plan	8/12 (67%)
13. Facilitate goal setting	3/9 (33%)
14. Prompt review of set goals	15/28 (54%)
15. Prompt self-recording	4/6 (67%)
16. Advise on changing routines	2/4 (50%)
17. Advise on environmental restructuring	4/6 (67%)
18. Advise on avoidance of cues for smoking	2/2 (100%)
19. Set graded tasks	0/4 (0%)
20. Advise on stop-smoking medication	32/34 (94%)
21. Advise on/facilitate use of social support	2/13 (15%)
22. Ask about experiences of stop smoking medications that the smoker is using	22/30 (73%)
23. Give options for additional/later support	3/7 (43%)
24. Emphasize choice	2/7 (29%)
25. Build general rapport	22/23 (96%)
26. General practitioner communication approaches	13/13 (100%)
27. Explain expectations regarding treatment programme	9/10 (90%)
28. Offer/direct towards appropriate written materials	7/25 (28%)
29. Information gathering and assessment	12/12 (100%)
30. Provide reassurance	8/13 (62%)

### 4. Delivery of BCTs not included in the manual (*i.e*., additional content)

In Service 1, sessions contained a mean total of 21 BCTs (SD 5; range: 8 to 27), of which 12 (57%; SD 4.8; range: 3 to 21) were not manual-specified. In Service 2, sessions contained a mean of 24 BCTs in total (SD 4.6, range: 12 to 31), of which 12 (50%; SD 3.17, range: 6 to 18) were not included in the treatment manual (Table 1; Additional file 1). Across both sets of transcripts (n = 34), the BCTs most frequently delivered as additional content were: ‘provide feedback on performance’ (n = 34, 100%) and ‘provide normative information on others’ experiences’ (n = 30, 88%) (Additional file 4).

## Discussion

Behaviour change techniques delivered in practice could be reliably coded, and this could be used to assess fidelity to treatment manuals in routine clinical practice. Behavioural support delivered by two English Stop-Smoking Services contained on average 66% of the BCTs specified in service treatment manuals, indicating that a third of the recommended service content was typically not delivered. General consensus indicates that 80% to 100% integrity to manual represents ‘high’ fidelity of delivery, whereas <50% represents ‘low fidelity’ [16,31,32]. There was substantial variability in the extent of fidelity of delivery across sessions from both services. While 32% of all sessions from both services displayed ‘high fidelity,’ the remaining two-thirds displayed levels of fidelity classifiable as either ‘moderate’ (approximately 65% fidelity) or ‘low.’ The levels of fidelity found in the current study reflect those obtained in similar studies assessing fidelity of delivery of behaviour change interventions in other domains [23,24] and adds to a growing body of evidence illustrating the inconsistency with which behaviour change interventions are implemented.

Variation in the degree of fidelity of delivery was observed within and across both services according to individual practitioners, session types and BCTs. For example, post-quit sessions displayed the highest levels of fidelity in Service 1, but the lowest in Service 2. Average levels of fidelity for individual practitioners varied by 23%. This may be influenced by professional backgrounds, years of experience, levels of supervision and training received, which varies substantially across practitioners in NHS Services [33]. It has not yet been established whether more experienced intervention providers have higher fidelity of delivery, but factors known to influence fidelity are provider’s perceived acceptability and effectiveness of treatment [16,34]. Levels of fidelity of delivery of individual BCTs also varied substantially, from perfect fidelity (100%) to none (0%).

Session duration was not significantly associated with extent of fidelity. Insufficient time to deliver manual-specified content is therefore unlikely to be an important contributing factor for failures to deliver prescribed content in this area. However, time taken to deliver each BCT was not accounted for in analyses. It is possible that some complex BCTs, such as ‘barrier identification and problem solving,’ take longer to deliver than BCTs such as ‘provide reassurance.’ Such variation across BCTs may have in part influenced the relationship between overall observed fidelity and session duration.

This widespread variability in fidelity of delivery allows for the identification of particularly problematic areas of intervention implementation and service provision in each service. Identifying those specific practitioners, types of sessions, and individual BCTs for which fidelity is lowest allows for the establishment of specific training needs to be targeted in future training and improvement guidelines. This in turn allows for more efficient, tailored use of training and development resources, and contributes to improvements in the design and implementation of more effective interventions. Some BCTs that were included in the manual and are known to be significantly associated with improved CO-validated quit outcomes [25], were delivered with low fidelity [*e.g*., ‘advise on changing routines’ (50%) and ‘advise on use of social support’ (15%)]. If component BCTs that are shown to be effective in research trials are to subsequently improve quit outcomes in clinical practice, health professionals delivering interventions must first adopt these BCTs routinely in practice [9].

The variations in the fidelity of delivery of the content of behavioural support found in this study represent one potential factor explaining existing variation in successful quit outcomes within and across English Stop-Smoking Services [28]. On average, half of all delivered content in both services was not manual-specified. We do not know whether delivery of these additional BCTs adds to effectiveness of, or dilutes, the impact of the manual-specified BCTs. It certainly increases variance in the delivery of the intervention and reduces the consistency in the content of support provided across sessions. Attempts to establish associations between the content of behavioural support specified in treatment manuals and quit outcomes cannot be accurately achieved unless the additional content delivered is first identified and accounted for in analyses. A review of audit and feedback interventions found ‘additional’ BCTs present in 86% of studies examined, which in turn hampered evidence synthesis and evaluation [35]. In the present study, of the additionally delivered BCTs, some featured consistently in all sessions despite not being prescribed in either service’s manual (*e.g*., ‘provide feedback on performance’), and others such as ‘boost motivation and self-efficacy’ have been shown to be effective [25]. It is possible that practitioners recognize the value of these BCTs, or that they are easier to deliver routinely or intuitively. If research evidence suggests such BCTs contribute to treatment success, they should be considered for inclusion in treatment manuals.

The taxonomy of smoking cessation BCTs demonstrated high reliability when applied to coding treatment manuals and session transcripts. It provided a consistent, common language by which to compare the content of manuals and sessions, and in turn quantify fidelity of delivery. The taxonomy therefore represents a suitable, systematic method by which the fidelity of smoking cessation behavioural support interventions may be assessed. It has been shown that novice coders may be reliably trained to code the content of treatment manuals and session transcripts using the taxonomy [27]. The taxonomy may therefore serve as a potentially feasible tool for service monitoring and evaluation. Taxonomies are available for other health behaviours, such as physical activity and healthy eating [36], alcohol use [37]; and a comprehensive non-behaviour specific taxonomy of BCTs is currently being developed [38]. Whether these taxonomies may be applied as tools for assessing fidelity of delivery of behaviour change interventions in these other behavioural domains is yet to be established.

This study raises the issue of the extent to which treatment manuals are fit for purpose. The evidence base for the BCTs in the services’ manuals was not assessed, nor was the extent to which manuals are clearly written and conform to training standards and national guidelines. This is not only necessary for interpreting results of fidelity assessments but also for comparing the quality of services provided, since both the planned content and the extent to which content is delivered are essential aspects of assessing the quality and hence likely impact of a service. For instance, the post-quit sessions delivered in Service 2 had an average lower percentage fidelity (56%) than those delivered in Service 1 (69%). However, the post-quit manual from Service 2 contained more BCTs (17) than that from Service 1 (10). The mean number of BCTs delivered per post-quit session in Service 2 was higher than that in Service 1 (approximately 10 vs. approximately 7 BCTs, respectively). Therefore, although fidelity appears to be poorer in Service 2, the post-quit sessions may in fact have potentially been more effective in helping clients successfully quit, as a higher number of techniques were delivered. This raises the question as to whether 100% fidelity is necessary to produce desired treatment outcomes [16]. Combining an analysis of the extent to which manuals are based on good evidence with an assessment of fidelity will give a more comprehensive assessment of delivery and stronger evidence of intervention quality than considering either evidence or fidelity on its own.

The question of whether 100% fidelity of intervention delivery is a desirable aim is under debate [16]. Strict adherence to treatment manuals may be detrimental to therapeutic interactions, as not all content specified in manuals will be relevant to all the individual needs and concerns of intervention recipients [39,40]. The delivery of additional, non-manual specified BCTs may be one means by which practitioners are tailoring the content of support provided to client needs and are increasing flexibility in their practice. Furthermore, the manuals from both services contained a high number of BCTs, which may not always be feasible or appropriate to deliver in practice. However, manuals are essential to maintaining a degree of consistency and standards in service provision. Some argue in favour of a middle ground in which core, prescribed intervention components are delivered with a degree of additional flexibility and tailoring in how content is provided. Such an approach does not compromise fundamental treatment integrity, and offers a potentially more feasible, realistic and beneficial model of treatment delivery [16,40].

Limitations of the current study firstly include the sample size of only two services, which means that these findings may not reflect all sessions delivered by practitioners, other services or behavioural support provided in contexts other than the English Stop-Smoking Services. In addition to assessing fidelity in terms of presence or absence of BCTs, it would be a step forward to establish a method for also assessing the quality with which BCTs are delivered. An additional key question is whether fidelity is associated with quit outcomes. Interventions implemented with higher levels of treatment fidelity have been shown to be associated with better treatment outcomes than those with poor fidelity in other areas [41]. However, the presently examined sample of services had high and average success rates respectively but similar levels of fidelity; the extent to which differences in fidelity may help explain variance in quit outcomes needs to be examined in future research with a representative sample. Audio recording was used rather than video recording, as it is less intrusive, more feasible and economical. Since all BCTs in the taxonomy require some degree of verbalisation (*e.g*., ‘advise on,’ ‘facilitate,’ ‘offer’)*,* video recording is unlikely to substantially add information in terms of content delivered. Since video recording is more intrusive, it is more likely to interfere with routine practice as a result of social desirability or demand characteristics. Nonetheless, practitioners were aware that their sessions were being audio recorded and may thus have been susceptible to demand characteristics and attempted to improve their practice under observation. Therefore, these sessions may not be representative of typical practice. However, these sessions are likely to represent a ‘best case scenario,’ and therefore over-estimate rather than under-estimate fidelity of delivery.

## Conclusions

The degree to which smoking cessation behavioural support interventions are implemented in routine clinical practice according to manual specifications can be reliably assessed. A preliminary analysis of service delivery in two English Stop-Smoking services demonstrated that manual-specified content, including numerous evidence-based BCTs, was not implemented with high fidelity. Manuals represent one potential tool for bridging the gap between evidence-base and practice in the implementation chain, as does training to implement those manuals. The present findings underline the general need to establish routine procedures for monitoring the fidelity with which behaviour change interventions are implemented in clinical practice, with a view to improving them where they are found short.

## Abbreviations

BCT: Behaviour change technique; CO: Carbon-monoxide; NHS: National Health Service; NCSCT: National Centre for Smoking Cessation and Training.

## Competing interests

FL has received travel funds and hospitality from Pfizer, who manufacture Champix. RW has undertaken research and consultancy for companies that develop and manufacture smoking cessation medications. RW has a share of a patent in a novel nicotine delivery device. SM has received travel funds and hospitality from Pfizer, who manufacture Champix. She has received fees for speaking at educational events sponsored by Pfizer. She has received research funds and consultancy payments from the Department of Health and the Department of Transport. CC declares no competing interests.

## Authors’ contributions

FL, SM, and RW conceived the study. FL and CC collected the audio recordings, transcripts, and service manuals and conducted preliminary analysis and coding of data. All authors participated in interpretation of the results. FL drafted the write-up, with input by SM and RW on subsequent drafts. All authors read and approved the final manuscript.

## Supplementary Material

Additional file 1 **Session characteristics and the proportion of BCTs specified in the treatment manuals delivered individual behavioural support sessions; presented by Stop Smoking Service and according to session type.***This table presents the session characteristics (i.e. duration, type) and the number of BCTs delivered with fidelity in each individual session.* Click here for file

Additional file 2 **BCTs included in the treatment manual from each Stop Smoking Service.***This table lists the BCTs identified in each session of the treatment manual from both services.* Click here for file

Additional file 3 **Proportion of behavioural support sessions each manual-specified BCT was delivered in according to session type (pre-quit, quit-day, post-quit), presented combined for both services.***This table presents the proportion of each type of session each manual-specified BCT was delivered in with fidelity.* Click here for file

Additional file 4 **Non-manual specified BCTs delivered in behavioural support sessions, presented according to session type and ranked according to frequency of transcripts featured in.***This table lists, in order of frequency, the BCTs most often delivered as additional, non-manual specified content, in sessions.* Click here for file

## References

[B1] Lancaster T Stead LF Individual behavioural counselling for smoking cessation Cochrane Database Syst Rev 2005 CD001292 15846616 10.1002/14651858.CD001292.pub2

[B2] Stead LF Lancaster T Group behaviour therapy programmes for smoking cessation Cochrane Database Syst Rev 2005 CD001007 15846610 10.1002/14651858.CD001007.pub2

[B3] Stead L Perera R Lancaster T Telephone counseling for smoking cessation Cochrane Database Syst Rev 2006 2005 CD002850 10.1002/14651858.CD002850.pub2 16855992

[B4] Michie S Churchill S West R Identifying evidence-based competences required to deliver behavioural support for smoking cessation Ann Behav Med 2011 41 1 59 70 10.1007/s12160-010-9235-z 20936389

[B5] West R Stapleton J Clinical and public health significance of treatments to aid smoking cessation Eur Respir Rev 2008 17 110 199 204 10.1183/09059180.00011005

[B6] Lorencatto F West R Michie S Identifying evidence-based behaviour change techniques for smoking cessation in pregnancy Nicotine & Tobacco Research 2012 14 9 1019 1026 10.1093/ntr/ntr324 22318690

[B7] Raw M Regan S Rigotti NA McNeill A A survey of tobacco dependence treatment services in 36 countries Addiction 2009 104 2 279 87 10.1111/j.1360-0443.2008.02443.x 19149825 PMC2756020

[B8] Ferguson J Bauld L Chesterman J Judge K The English smoking treatment services: one-year outcomes Addiction 2005 100 2 59 69 15755262 10.1111/j.1360-0443.2005.01028.x

[B9] Eccles M Armstrong D Baker R Cleary K Davies H Davies S An implementation research agenda Implement Sci 2009 4 18 1 7 19351400 10.1186/1748-5908-4-18 PMC2671479

[B10] Wilson G Manual-based treatments: the clinical application of research findings Behav Res Ther 1996 34 295 314 10.1016/0005-7967(95)00084-4 8871362

[B11] Wallace L Ranson M Treatment manuals: Use in treatment of bulimia nervosa Behav Res Ther 2011 49 815 820 10.1016/j.brat.2011.09.002 21939960

[B12] Cukrowicz K Timmons K Sawyer K Caron K Gummelt H Joiner T Improved treatment outcome associated witht he shift to empirically supported treatments in an outpatient clinic is maintained over a ten-year period Professional Psychology: Research and Practice 2011 42 145 152

[B13] Croghan E Local Stop Smoking Services: Service delivery and monitoring guidance 2011/2012 2011 Department of Health Accessed on 11-10-2012. Retrieved from: http://www.dh.gov.uk/prod_consum_dh/groups/dh_digitalassets/documents/digitalasset/dh_125939.pdf

[B14] West R McNeill A Raw M National smoking cessation guidelines for health professionals: an update Thorax 2000 55 12 987 999 10.1136/thorax.55.12.987 11083883 PMC1745657

[B15] Brose LS McEwen A West R Does it matter who you see to help you stop smoking? short-term quit rates across specialist stop smoking practitioners in England Addiction 2012 107 11 2029 2036 online first 9.5.2012. in press 10.1111/j.1360-0443.2012.03935.x 22571648

[B16] Borrelli B The assessment, monitoring and enhancement of fidelity in public health clinical trials Journal of Public Health Dentistry 2011 71 s1 52 63 10.1111/j.1752-7325.2011.00233.x PMC3074245 21499543

[B17] Bellg A Borrelli B Resnick B Hecht J Minicucci DS Ory M Ogedegbe G Orwig D Ernst D Czajkowski S Enhancing treatment fidelity in health behaviour change studies: best practices and recommendations from the NIH behaviour change consortium Health Psychology 2004 22 5 443 451 15367063 10.1037/0278-6133.23.5.443

[B18] Dusenbury L Brannigan R Falco M Hansen WB A review of research on fidelity of implementation: implications for drug abuse prevention in school settings Health Education Research: Theory and Practice 2003 18 2 237 256 10.1093/her/18.2.237 12729182

[B19] Boutron I Moher D Altman D Scultz K Ravaud P Extending the CONSORT statement to randomized trials of Non-pharmacologic treatment: explanation and elaboration Annals of Internal Medicine 2008 148 295 309 18283207 10.7326/0003-4819-148-4-200802190-00008

[B20] Moncher F Prinz R Treatment fidelity in outcome studies Clinical psychology review 1991 11 247 266 10.1016/0272-7358(91)90103-2

[B21] Dane A Schnieder B Program integrity in primary and early secondary prevention: are implementation effects out of control? Clinical Psychology Review 1998 18 22 45 10.1016/s0272-7358(97)00043-3 9455622

[B22] Hardeman W Michie S Fanshawe T Prevost T Mcloughlin K Kinmonth AL Fidelity of a physical activity intervention: predictors and consequences Psychology and Health 2008 23 1 11 24 10.1080/08870440701615948 25159904

[B23] Tober G Clyne W Finnegan O Farrin A Russell I Validation of a scale for rating the delivery of psycho-social treatments for alcohol dependence and misuse: the UKATT process rating scale (PRS) Alcohol and Alcoholism 2008 43 6 675 682 10.1093/alcalc/agn064 18818190

[B24] Michie S Walia A Hyder N West R Development of a taxonomy of behaviour change techniques used in individual behavioural support for smoking cessation Addictive behaviours 2010 36 4 315 319 10.1016/j.addbeh.2010.11.016 21215528

[B25] West R Walia A Hyder N Michie S Behaviour change techniques used by the English stop smoking services and their associations with short-term quit outcomes Nicotine & Tobacco Research 2010 12 7 742 747 10.1093/ntr/ntq074 20478957

[B26] Michie S Churchill S West R Identifying evidence-based competences required to deliver behavioural support for smoking cessation Annals of Behavioural Medicine 2011 41 49 70 10.1007/s12160-010-9235-z 20936389

[B27] Lorencatto F West R Seymour N Kaplunov E Michie S Developing a method for specifying components of behaviour change interventions delivered in practice: the example of smoking cessation Journal of Consulting and Clinical Psychology 2013 In Press 10.1037/a0032106 23506465

[B28] NHS Information Centre Statistics on NHS Stop Smoking Services: England, April 2010 to December 2011. NHS IC 2011 Accessed on 10-05-2012. Retrieved from: https://catalogue.ic.nhs.uk/publications/public-health/smoking/stat-stop-smok-serv-eng-apr-dec-11-q3/stat-stop-smok-eng-2011-12-q3-app.pdf

[B29] West R Lorencatto F Michie S Churchill S Willis N McEwen A NCSCT Training Standards: Learning outcomes for training stop smoking specialists 2010 NHS Centre for Smoking Cessatio and Training, Department of Health UK Available at: http://www.ncsct.co.uk/usr/pub/NCSCT%20Training%20Standard.pdf

[B30] Steinijans V Diletti E Bomches B Greis C Solleder P Interobserver agreement: Cohen’s kappa coefficient does not necessarily reflect the percentage of patients with congruent classifications Int Journal Clinical Pharmacological Therapy 1997 35 3 93 5 9088995

[B31] Noell G Gresham F Gansle K Does treatment integrity matter? A preliminary investigation of instructional implementation and mathematical performance Journal of Behavioural Education 2002 22 51 67

[B32] Holcombe A Wolery M Synder E Effects of two levels of procedural fidelity with constant time delay on children’s leraning Journal of Behavioural Education 1994 4 49 73 10.1007/BF01560509

[B33] McDermott M Thomson H West R Kenyon J McEwen A Translating evidence-based guidelines into practice: a survey of practices of commissioners and managers of the English Stop Smoking Services BMC Health Services Research 2012 12 121 1 8 22621715 10.1186/1472-6963-12-121 PMC3444944

[B34] Borrelli B Hecht J Papandonatos G Emmons J Tatewosian L Abrams D Smoking cessation counseling in the home. Attitdues, beliefs, and behaviours of home healthcare nurses American Journal Preventative Medicine 2001 21 4 272 7 10.1016/S0749-3797(01)00369-5 11701297

[B35] Gardner B Whittington C McAteer J Eccles M Michie S Using theory to synthesise evidence from behaviour change interventions: the example of audit and feedback Social Science Medicine 2010 70 10 1618 25 10.1016/j.socscimed.2010.01.039 20207464

[B36] Michie S Ashford S Sniehotta FF Dombrowski SU Bishop A French DP A refined taxonomy of behaviour change techniques to help people change their physical activity and healthy eating behaviours – the CALO-RE taxonomy Psychology & Health 2011 26 11 1479 1498 10.1080/08870446.2010.540664 21678185

[B37] Michie S Whittington C Hamoudi Z Zarani F Tober G West R Behaviour change techniques to reduce excessive alcohol use and their associations with outcome Addiction 2012 107 8 1431 1440 10.1111/j.1360-0443.2012.03845.x 22340523

[B38] Michie S Abraham C Eccles M Francis J Hardeman W Johnston M Strengthening evaluation and implementation by specifying components of behaviour change interventions: a study protocol Implementation Science 2011 6 10 10.1186/1748-5908-6-10 PMC3041694 21299860

[B39] Leventhal H Friedman M Does establishing fidelity of treatment help in understanding treatment efficacy? Health Psychology 2004 23 5 452 456 15367064 10.1037/0278-6133.23.5.452

[B40] Kendall P Gosch E Furr J Sood E Flexibility within fidelity Journal A, Acad Child Adolescent Psychiatry 2008 47 9 987 93 10.1097/CHI.0b013e31817eed2f 18714195

[B41] Durlak J DuPre E Implementation matters: a review of research on the influence of implementation on program outcomes and the factors affecting implementation American Journal of Community Psychology 2008 41 3 327 50 10.1007/s10464-008-9165-0 18322790

