# Supplementary material for: Assessing fidelity of delivery of smoking cessation behavioural support in practice
Source: Implement Sci. 2013 Apr 4;8:40. doi: 10.1186/1748-5908-8-40 (PMC3622616; doi:10.1186/1748-5908-8-40)
Supplement: Additional file 1 — Session characteristics and the proportion of BCTs specified in the treatment manuals delivered individual behavioural support sessions; presented by Stop Smoking Service and according to session type.This table presents the session characteristics (i.e. duration, type) and the number of BCTs delivered with fidelity in each individual session. [file 1748-5908-8-40-S1.pdf]

Additional File 1: Session characteristics and the proportion of BCTs specified in the treatment manuals delivered individual behavioural support sessions; presented by Stop Smoking Service and according to session type.

Service ID	Session ID (Service No./ Transcript No.)	Practitioner ID (Service No./ Transcript No.)	Session type (1=pre-quit; 2=quit-day; 3=post-quit	Duration (Min.Sec)	Number of BCTs in Manual (according to session type)	Number of manual specified BCTs in Session (%)	Total Number of BCTs in session	Number non-manual specified BCTs in session (% total BCTs)
Service 1 (n=21 transcripts)								
S01	S01T01	S01P01	3	10.59	10	8 (80%)	14	6 (43%)
S01	S01T02	S01P01	3	9.36	10	6 (60%)	17	11 (65%)
S01	S01T03	S01P01	3	8.09	10	8 (80%)	21	13 (61%)
S01	S01T04	S01P01	3	12.27	10	9 (90%)	22	13 (59%)
S01	S01T08	S01P02	3	5.01	10	5 (50%)	8	3 (34%)
S01	S01T10	S01P02	3	5.15	10	6 (60%)	11	5 (45%)
S01	S01T11	S01P02	3	7.19	10	7 (70%)	21	14 (66%)
S01	S01T07	S01P03	2	16.46	8	6(75%)	27	21 (78%)
S01	S01T09	S01P03	1	36.36	13	9 (69%)	25	16 (64%)
S01	S01T14	S01P03	3	17.00	10	5(50%)	22	17 (77%)
S01	S01T15	S01P03	3	11.01	10	8 (80%)	22	14 (64%)
S01	S01T16	S01P03	3	18.21	10	8 (80%)	23	15 (65%)
S01	S01T06	S01P04	3	16.57	10	4 (40%)	23	19 (82%)
S01	S01T12	S01P04	2	20.32	8	4 (50%)	19	15 (79%)
S01	S01T13	S01P04	1	22.19	13	9(69%)	17	8 (47%)
S01	S01T14	S01P04	1	29.35	13	5 (38%)	20	15 (75%)
S01	S01T18	S01P04	3	13.51	10	8 (80%)	26	18 (69%)
S01	S01T21	S01P04	1	26.45	13	7 (54%)	25	18(72%)
S01	S01T05	S01P05	3	5.45	10	5 (50%)	14	9 (64%)
S01	S01T19	S01P05	3	21.50	10	9(90%)	22	13(59%)
S01	S01T20	S01P05	3	15.14	10	8(80%)	24	16 (67%)
Service 2(n=13 transcripts)								
S02	S02T08	S02P01	3	5.17	17	6 (35%)	12	6 (50%)
S02	S02T10	S02P01	3	14.22	17	11(64%)	20	9 (45%)
S02	S02T11	S02P01	3	8.01	17	8 (47%)	20	12 (60%)
S02	S02T06	S02P02	2	20.17	21	16(76%)	27	11 (41%)
S02	S02T13	S02P02	3	11.45	17	11(64%)	23	12 (52%)
S02	S02T01	S02P03	1	12.10	12	8 (67%)	23	15 (65%)
S02	S02T02	S02P03	1	5.47	12	10 (83%)	18	8 (44%)
S02	S02T03	S02P03	1	17.48	12	10 (83%)	25	15 60%)
S02	S02T09	S02P03	3	8.09	17	11 (64%)	22	11 (50%)
S02	S02T04	S02P04	1	15.46	12	8 (67%)	26	18 (69%)
S02	S02T05	S02P04	2	13.15	21	18(85%)	31	13(42%)
S02	S02T07	S02P04	3	12.31	17	9 (53%)	23	12 (52%)
S02	S02T12	S02P04	3	18.02	17	11 (64%)	21	10 (48%)
