# Supplementary material for: Assessing fidelity of delivery of smoking cessation behavioural support in practice
Source: Implement Sci. 2013 Apr 4;8:40. doi: 10.1186/1748-5908-8-40 (PMC3622616; doi:10.1186/1748-5908-8-40)
Supplement: Additional file 2 — BCTs included in the treatment manual from each Stop Smoking Service.This table lists the BCTs identified in each session of the treatment manual from both services. [file 1748-5908-8-40-S2.pdf]

Additional File 2: BCTs included in the treatment manual from each Stop Smoking Service.

Note: *BCTs presented in bold are present in both manuals in the relevant sub-section*

Session Type	BCTs in manual from Service 1	BCTs in manual from Service 2
**Pre-quit**	- **Measure and explain purpose of CO monitoring**<br>- **Action planning**<br>- **Advise on stop smoking medications**<br>- **Information gathering and assessment**<br>- **Explain expectations regarding the treatment programme**<br>- **Offer/direct towards appropriate written materials**<br>- Build rapport<br>- Advise on use of social support<br>- Prompt commitment from the client there and then<br>- Advise on environmental restructuring<br>- General practitioner communication approaches<br>- Provide reassurance<br>- Goal setting	- **Measure and explain purpose of CO monitoring**<br>- **Action planning**<br>- **Advise on stop smoking medications**<br>- **Information gathering and assessment**<br>- **Explain expectations regarding the t**<br>- **Offer/direct towards appropriate written materials**<br>- Identify reasons for wanting/not wanting to stop smoking<br>- Facilitate relapse prevention and coping<br>- Prompt review of set goals<br>- Prompt self-recording<br>- Advise on changing routines<br>- Set graded tasks
**Quit-day**	- **Measure and explain purpose of CO monitoring**<br>- **Facilitate relapse prevention and coping**<br>- **Ask about experiences of stop-smoking medications the client is currently using**<br>- **Build rapport**<br>- **Information gathering and assessment**<br>- Goal setting<br>- Prompt review of set goals<br>- Provide options for additional/later support	- **Measure and explain purpose of CO monitoring**<br>- **Facilitate relapse prevention and coping**<br>- **Ask about experiences of stop-smoking medications the client is currently using**<br>- **Build rapport**<br>- **Information gathering and assessment**<br>- Boost motivation and self-efficacy<br>- Prompt commitment from the client there and then<br>- Strengthen ex-smoker identity<br>- Identify reasons for wanting/not wanting to stop smoking<br>- Distract from motivation to engage in behaviour<br>- Barrier identification and problem solving<br>- Action planning<br>- Prompt self-recording<br>- Advise on environmental-restructuring<br>- Advise on avoidance of cues for smoking<br>- Advise on stop smoking

		medications<br>- Advise on use of social support<br>- Explain expectations regarding the treatment programme<br>- Offer/direct towards appropriate written materials<br>- General practitioner communication approaches<br>- Provide reassurance
**Post-quit**	- **Provide rewards contingent on successfully stopping smoking**<br>- **Provide rewards contingent on effort or progress**<br>- **Measure and explain purpose of CO monitoring**<br>- **Facilitate relapse prevention and coping**<br>- **Advise on stop smoking medications**<br>- **Ask about experiences of stop smoking medications that the client is currently using**<br>- **Prompt review of set goals**<br>- Information gathering and assessment<br>- Build rapport<br>- Offer/direct towards appropriate written materials	- **Provide rewards contingent on successfully stopping smoking**<br>- **Provide rewards contingent on effort or progress**<br>- **Measure and explain purpose of CO monitoring**<br>- **Facilitate relapse prevention and coping**<br>- **Advise on stop smoking medications**<br>- **Ask about experiences of stop smoking medications that the client is currently using**<br>- **Prompt review of set goals**<br>- Provide information on the consequences of smoking and smoking cessation<br>- Prompt commitment from the client there and then<br>- Strengthen ex-smoker identity<br>- Barrier identification and problem solving<br>- Goal setting<br>- Advise on use of social support<br>- Provide options for additional/later support<br>- Emphasise choice<br>- General practitioner communication approaches<br>- Provide reassurance
