# Supplementary material for: Assessing fidelity of delivery of smoking cessation behavioural support in practice
Source: Implement Sci. 2013 Apr 4;8:40. doi: 10.1186/1748-5908-8-40 (PMC3622616; doi:10.1186/1748-5908-8-40)
Supplement: Additional file 3 — Proportion of behavioural support sessions each manual-specified BCT was delivered in according to session type (pre-quit, quit-day, post-quit), presented combined for both services.This table presents the proportion of each type of session each manual-specified BCT was delivered in with fidelity. [file 1748-5908-8-40-S3.pdf]

Additional File 3: Proportion of behavioural support sessions each manual-specified BCT was delivered in according to session type (pre-quit, quit-day, post-quit), presented combined for both services.

BCT Label	No. pre-quit sessions delivered in according to pre-quit manual specification	No. quit-day sessions delivered in according to quit-day manual specification	No. post-quit sessions delivered in according to post-quit manual specification	Total No. sessions BCT delivered in according to manual specification (max n=34)
Provide rewards contingent on successfully stopping smoking	-*	-	13/22	13/22 (59%)
Provide rewards contingent on effort or progress	-	-	18/22	18/22 (82%)
Identify reasons for wanting/not wanting to stop smoking	4/4 (100%)	1/ 2 (50%)	4/7	9/13 (69%)
Measure CO and explain purpose of CO monitoring	8/8 (100%)	4/4 (100%)	18/22 (82%)	30/34 (88%)
Facilitate relapse prevention and coping	0/4 (0%)	1/4 (25%)	9/22 (41%)	10/30 (33%)
Facilitate action planning/ develop treatment plan	6/8 (75%)	2/2 (100%)	-	8/10 (80%)
Facilitate goal setting	-	2/2 (100%)	1/7 (14%)	3/9 (33%)
Prompt review of set goals	0/4 (0%)	0/2 (0%)	15/22 (68%)	15/28 (53%)
Prompt self-recording	3/4 (75%)	1/2 (50%)	-	4/6 (67%)
Advise on changing routine	2/4 (50%)	-	-	2/4 (50%)
Advise on environmental restructuring	2/4 (50%)	2/2 (100%)	-	4/6 (67%)
Set graded tasks	0/4 (0%)	-	-	0/4 (0%)
Advise on stop smoking medication	8/8 (100%)	4/4 (100%)	20/22 (91%)	32/34 (94%)
Ask about experience of stop smoking medications the smoker is using	0/4 (0%)	4/4 (100%)	18/22 (82%)	22/30 (73%)
Build rapport	4/4 (100%)	2/2 (100%)	14/15 (93%)	20/21 (95%)
Explain expectations regarding the treatment programme	8/8 (100%)	1/2 (50%)	-	9/10 (90%)
Offer/direct towards appropriate written materials	5/8 (63%)	2/2 (100%)	0/15 (0%)	7/25 (28%)
