# Supplementary material for: Assessing fidelity of delivery of smoking cessation behavioural support in practice
Source: Implement Sci. 2013 Apr 4;8:40. doi: 10.1186/1748-5908-8-40 (PMC3622616; doi:10.1186/1748-5908-8-40)
Supplement: Additional file 4 — Non-manual specified BCTs delivered in behavioural support sessions, presented according to session type and ranked according to frequency of transcripts featured in.This table lists, in order of frequency, the BCTs most often delivered as additional, non-manual specified content, in sessions. [file 1748-5908-8-40-S4.pdf]

Additional File 4: Non-manual specified BCTs delivered in behavioural support sessions, presented according to session type and ranked according to frequency of transcripts featured in.

BCT label	Number of transcripts featured in (% of total)
Pre-quit transcripts (max 8)	
‘provide feedback on progress and performance’	8 (100%)
‘provide normative information on other’s experiences’	8 (100%)
‘provide options for additional/later support’	8 (100%)
‘build rapport’	8 (100%)
‘general practitioner communication approaches’	8 (100%)
‘provide reassurance’	8 (100%)
‘explain how nicotine dependence develops’	7 (87.5%)
‘boost motivation and self-efficacy’	6 (75%)
‘emphasise choice’	6 (75%)
‘provide information on the consequences of smoking and smoking cessation’	5 (62.5%)
‘barrier identification and problem solving’	5 (62.5%)
‘reflective listening’	5 (62.5%)
‘goal setting’	4 (50.0%)
‘Advise on conserving mental resources’	4 (50.0%)
‘Ask about current stop smoking medications the smoker is using’	4 (50.0%)
‘strengthen ex-smoker identity’	3 (37.5%)
‘advise on avoidance of cues for smoking’	3 (37.5%)
‘explain expectations regarding the treatment programme’	3 (37.5%)
‘provide rewards contingent on effort or progress’	2 (25.0%)
‘advise on changing routine’	2 (25.0%)
‘tailor interactions appropriately’	2 (25.0%)
‘offer/direct towards appropriate written materials’	2 (25.0%)
‘prompt behavioural substitution’	2 (25.0%)
‘distract from motivation to engage in behaviour’	1 (12.5%)
‘facilitate restructuring of social life’	1 (12.5%)
‘facilitate relapse prevention and coping’	1 (12.5%)
Quit-day transcripts (max 4)	
‘provide feedback on progress and performance’	4 (100%)
‘provide normative information on other’s experiences’	4 (100%)
‘emphasise choice’	4 (100%)
‘provide information on the consequences of smoking and smoking cessation’	3 (75%)
‘advise on changing routine’	3 (75%)
‘explain how nicotine dependence develops’	3 (75%)
‘reflective listening’	3 (75%)
‘provide rewards contingent on effort or progress’	2 (50%)
‘goal setting’	2 (50%)
‘prompt review of set goals’	2 (50%)
‘explain expectations regarding the treatment programme’	2 (50%)
‘boost motivation and self-efficacy’	2 (50%)
‘create or reinforce negative associations’	2 (50%)
‘barrier identification and problem solving’	2 (50%)
‘provide options for additional/later support’	2 (50%)
‘build rapport’	2 (50%)
‘general practitioner communication approaches’	2 (50%)
‘provide reassurance’	2 (50%)

‘provide information on withdrawal symptoms’	2 (50%)
‘advise on use of social support’	1 (25%)
‘advise on avoidance of cues for smoking’	1 (25%)
‘advise on conserving mental resources’	1 (25%)
‘advise on environmental restructuring’	1 (25%)
‘prompt self-recording’	1 (25%)
‘action planning/develop treatment plan’	1 (25%)
‘help identify reasons for wanting/not wanting to stop smoking’	1 (25%)
‘strengthen ex-smoker identity’	1 (25%)
‘prompt self-reward’	1 (25%)
‘facilitate restructuring of social life’	1 (25%)
‘emphasise the importance of abrupt cessation’	1 (25%)
Post-quit transcripts (max = 22)	
‘provide feedback on progress and performance’	22 (100%)
‘information gathering and assessment’	22 (100%
‘boost motivation and self-efficacy’	20 (90.1%)
‘provide normative information on other’s experiences’	18 (90.0%)
‘reflective listening’	16 (72.7%)
‘general practitioner communication approaches’	15 (68.1%)
‘provide reassurance’	15 (68.1%)
‘strengthen ex-smoker identity’	14 (63.6%)
‘barrier identification and problem solving’	14 (63.6%)
‘explain how nicotine dependence develops’	13 (59.1%)
‘provide information on withdrawal symptoms’	10 (45.5%)
‘emphasise choice’	9 (40.1%)
‘provide information on the consequences of smoking and smoking cessation’	8 (36.4%)
‘advise on changing routine’	7 (31.8%)
‘build general rapport’	7 (31.8%)
‘advise on conserving mental resources’	6 (27.3%)
‘explain expectations regarding the treatment programme’	6 (27.3%)
‘help identify reasons for wanting/not wanting to stop smoking’	6 (27.3%)
‘create or reinforce negative associations’	6 (27.3%)
‘prompt behavioural substitution’	5 (22.7%)
‘emphasise the importance of abrupt cessation’	4 (18.2%)
‘distract from motivation to engage in behaviour’	4 (18.2%)
‘action planning/develop treatment plan’	4 (18.2%)
‘prompt self-reward’	4 (18.2%)
‘ask about stop smoking medications the client is currently using’	4 (18.2%)
‘advise on avoidance of smoking cues’	2 (9.09%)
‘facilitate restructuring of social life’	2 (9.09%)
‘prompt commitment from the client there and then’	2 (9.09%)
‘advise on use of social support’	2 (9.09%)
‘advise on environmental restructuring	1 (4.55%)
‘prompt self-recording’	1 (4.55%)
‘goal setting’	1 (4.55%)
‘offer/direct towards appropriate written materials’	1 (4.55%)
‘tailor interactions appropriately	1 (4.55%)
‘advise on weight control’	1 (4.55%)
‘set graded tasks’	1 (4.55%)
